# CD28 Gene Polymorphisms in the Promoter Region Are Associated with Transfusion Reactions: A Functional Study

**DOI:** 10.3390/jcm10040871

**Published:** 2021-02-20

**Authors:** Ying-Hao Wen, Wei-Tzu Lin, Wei-Ting Wang, Ding-Ping Chen

**Affiliations:** 1 Department of Laboratory Medicine, Linkou Chang Gung Memorial Hospital, Taoyuan 33305, Taiwan; b9209011@cgmh.org.tw (Y.-H.W.); berry0908@cgmh.org.tw (W.-T.L.); s1223@cgmh.org.tw (W.-T.W.); 2 Graduate Institute of Clinical Medical Sciences, College of Medicine, Chang Gung University, Taoyuan 33302, Taiwan; 3 Department of Medical Biotechnology and Laboratory Science, Chang Gung University, Taoyuan 33302, Taiwan; 4 Graduate Institute of Biomedical Sciences, College of Medicine, Chang Gung University, Taoyuan 33302, Taiwan

**Keywords:** CD28, promoter, single nucleotide polymorphism, functional study

## Abstract

Transfusion reactions are mainly induced by the interaction of an antigen and antibody. However, transfusion reactions still occur with the implementing of crossmatching and usage of pre-storage leukoreduced blood products. The roles of CD28 and CTLA4 gene polymorphisms in transfusion reaction have been shown, and subjects with certain single nucleotide polymorphisms (SNPs) of the CD28 or CTLA4 gene had a significantly higher risk of transfusion reactions. In total, 40 patients with transfusion reactions after receiving pre-storage leukoreduced blood products were enrolled in this study. We focused on the SNPs located in the CD28 promoter region (rs1879877, rs3181096, rs3181097, and rs3181098) to find out the significant SNP. A luciferase reporter assay was used to investigate the expression level of protein affected by promoter SNP variation. We found that the polymorphism of rs3181097 was associated with transfusion reactions (*p* = 0.003 in additive model and *p* = 0.015 in dominant model). Consequently, we investigated the biological function in the CD28 promoter polymorphisms (rs1879877 G > T, rs3181096 C > T, rs3181097 G > A, and rs3181098 G > A) by using dual-spectral luciferase reporter assay. The results showed that the ex-pression level of CD28 was decreased under the effect of rs3181097 with A-allele. This suggested that rs3181097 may regulate immune response through decreasing CD28 protein expression and then lead to development of transfusion reactions.

## 1. Introduction

Blood transfusion is a procedure that is used for correcting deficiencies of blood components. In patients with cancers, they may need blood transfusions because of internal bleeding, cancer cells affecting the kidney or spleen, or bone marrow dysfunction caused by chemotherapy and radiotherapy, which will lead to anemia or shortage of other blood components [1]. Transfusion reaction is an immune response where the compositions of blood products transfused into patients are attacked from the host immune cells [2]. It is more likely to occur in women with multiple pregnancies and persons with repeated transfusions, such as cancer patients [3,4]. Although transfusion reaction is mainly induced by the interaction of antigens and antibodies, transfusion reactions still occur with the implementing of crossmatching and usage of pre-storage leukoreduced blood products [5]. The roles of CD28 and CTLA4 gene polymorphisms in transfusion reactions have been shown, and subjects with certain single nucleotide poly-morphisms (SNPs) of CD28 or CTLA4 gene had a significantly higher risk of transfusion reactions [6,7].

T cells play a vital role in both cell-mediated and humoral immune responses [8]. In the pathway of T cell activation, major histocompatibility complexes expressed on antigen-presenting cells integrated with T cell receptors to provide the first signal, as well as costimulatory factors interacting with the B7 ligand to provide the secondary signal [9]. If the secondary signal does not provide a positive signal in T cell activation, the T cell will become anergic [10]. Therefore, costimulatory molecules play an important role in immune response regulation [11]. CD28 is one of the costimulatory molecules mainly providing a stimulating signal in T cell activation, and it is continuously expressed on T cells [12].

Genetic variation in the promoter sequence may affect the efficiency of transcription and the expression level of protein, and ultimately the specific physiological functions would be changed [13]. Several studies had indicated that the SNPs located in the promoter region of the CD28 gene were related to various autoimmune diseases and cancers. Such is the rs3181097; we found that it was associated with transfusion reaction [6], and it also had correlation to the clinical pathology of IgA nephropathy in Korean children [14]. Moreover, rs1879877 and rs3181096 were found to be related to type 1 diabetes in Tunisians [15]. Additionally, rs3181098 has been shown that it was associated with the risk of recurrent spontaneous abortion [16], the risk of cancer in the Polish population [17], and the recovery of CD4+ T cells after treatment of human immunodeficiency virus infection with antiretroviral therapy [18].

Herein, we firstly performed the case control study on the association of CD28 gene polymorphisms in the promoter region and transfusion reactions occurring after transfusing leukoreduced blood products. Then, to explore the mechanism of CD28 in inducing transfusion reactions, we conducted the functional study on aforementioned four interested SNPs in the promoter region of CD28 to determine whether these SNPs would affect the expression level of CD28.

## 2. Experimental Section

### 2.1. Subjects

There were 40 patients suffering from transfusion reactions after transfusing leukocyte-poor red blood cells (LPR) or leukocyte-poor platelets (LPP). Transfusion reactions were investigated and diagnosed by clinical pathologists of the Department of Laboratory Medicine according to the National Healthcare Safety Network (NHSN) Hemovigilance Module of CDC. The healthy control group contained 40 subjects without autoimmune diseases or cancer. The average age of the 40 healthy controls was 22.6 ± 1.1 years old; 26 of them were female and 14 were male. These subjects were enrolled from September 2019 to August 2020, and they all signed the written consent form.

### 2.2. DNA Extraction

The peripheral blood samples of patients and healthy controls were collected in the EDTA-coated vacuum tubes. A QIAamp DNA Mini Kit (Qiagen GmbH, Hilden, Germany) was used to extract the genomic DNA according to the manufacturer’s instructions. Then, the concentration and purity of DNA were estimated by measuring the optical density at 260 and 280 nm through UV spectrometer.

### 2.3. PCR Amplification and Sequencing

We focused on the SNPs located in the CD28 promoter region (rs1879877, rs3181096, rs3181097, and rs3181098). The CD28 promoter sequence was amplified with the forward primer 5′- GGG TGG TAA GAA TGT GGA TGA ATC-3′ and the reverse primer 5′-CAA GGC ATC CTG ACT GCA GCA-3′. The mixture for the PCR reaction contained 1 μL of sample DNA, 10 μL of HotStarTaq DNA Polymerase (Qiagen GmbH, Hilden, Germany), 12 μL of ddH2O, and 1 μL of 10 μM of each of the forward primer and reverse primer. These mixtures were received an initial denaturation at 95 °C for 3 min, followed by 30 cycles of 95 °C for 30 s, 58 °C for 30 s, and 72 °C for 2 min, and the final extension step was 72 °C for 10 min. The PCR products were sequenced by using ABI PRISM 3730 DNA analyzer (Ap-plied Biosystems, Foster City, CA). Due to the insufficient genomic DNA and unclear sequencing information, the SNP data were not all available.

### 2.4. Promoter-Reporter Constructs

The genomic region upstream of the CD28 transcription start site (TSS) was amplified with primers 5′- TAT GAGCTC AGC AGT TGG CCG TGC TGG TGG AAT -3′ and 5′- TTA TAA GCT TGG GTT CCA GCC CCT CCT CCC CGA -3′ from homozygous individuals for either one or the other allelic variant and cloned into pCRII Vector (Invitrogen, Life Technologies, Carlsbad, CA, USA). This construct was subsequently digested with SacI and HindIII to generate a fragment spanning the promoter region from nt −1232 to +200 (including the rs1879877, rs3181096, rs3181097, rs3181098 SNP) and transferred to the pNL1.1 [Nluc] expression vector (Promega). The SNPs were introduced in the expression constructs by site-directed mutagenesis using the Quick-Change mutagenesis kit (Strata-gene), and the primers as listed in Table 1 below.

### 2.5. Cell Culture and Transient Transfections

The K562 cells (1 × 106) were routinely cultured in 90% Roswell Park Memorial Institute (RPMI) 1640 medium supplemented with 10% fetal bovine serum, penicillin (50 U/mL), and streptomycin (50 μg/mL), and were transfected with 5 μg of allele-specific expression vector using LF2000 according to the manufacturer’s instructions. Then, 400 μL (2.5 × 105) K562 cells were transfected with 1 μg of either one of the constructs and 1 μg of the pGL 4.5 [Luc2/TK] vector (Promega) as an internal control for transfection efficiency using Lipofectamine 2000 (Invitrogen).

### 2.6. Luciferase Reporter Assay

**Figure 1:**
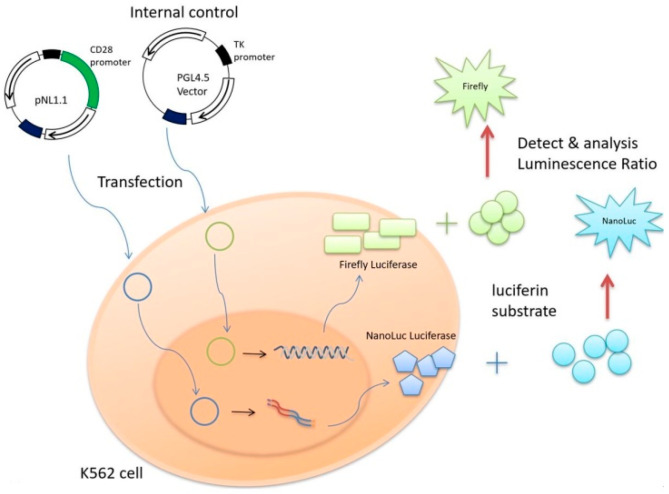
The schematic diagram of luciferase reporter assay.

Luciferase assay was carried out using the Luciferase Assay System (Nano-Glo^®^ Du-al-Luciferase^®^ Reporter Assay System, Promega) according to the manufacturer’s protocol. Twenty-four hours after transfection, cells were lysed with Passive Lysis Buffer (Promega), and luciferase activity was measured using a Nano-Glo^®^ Luciferase Assay System (Promega) and a GloMax Discover System (Promega). The PGL4.5 vector (Firefly luciferase) was used as an internal control to reduce the deviation of transfection efficiency caused by experimental error. The schematic drawing of the principle of the luciferase reporter assay is shown in Figure 1.

There were two vectors transfected into K562 cell. One was the pGL 4.5 vector, which was used as an internal control that translated the Firefly luciferase, while the four promoter-reporter constructs transferred to pNL1.1 expression vector and translated the NanoLuc luciferase. After culturing for 24 h, we analyzed the changing effect of CD28 promoter-reporter on luciferase activity by detecting the luminescence ratio of NanoLuc luciferase to Firefly luciferase.

### 2.7. Statistical Analysis

For the case control study, the Hardy–Weinberg equilibrium (HWE) was used to analyze the genetic variation at equilibrium in control group in order to reduce the bias. The chi-squared test and Fisher’s exact test were used to analyze the difference of allele frequency and genotype frequency between patients and healthy controls. These statistical data were calculated by SPSS 17.0. For a functional study, each sample was performed in duplicate and each experiment was independently repeated five times. All the results of luciferase assay from each promoter reporters were analyzed through the ratio of NanoLuc to Firefly. Then, the luminescence value of the wild type (Grs1879877Crs3181096Grs3181097Grs3181098) was assumed as a reference for relative light unit (RLU). The difference of RLU between wild type and each promoter reporters was calculated by independent-sample *t*-tests.

## 3. Results

There were 40 patients with transfusion reactions after transfusing LPR (21 cases) or LPP (19 cases), and 40 healthy controls were enrolled in the case control study. The characteristics of patients are listed in Table 2. Their average age was 54.7 years, and the gender ratio was 3:1 (female:male). Among these patients, most of them were O type blood (45%). Twenty-nine patients suffered from febrile non-hemolytic transfusion reactions (FNHTR), and 11 patients suffered from allergic reactions.

In Table 3, all SNPs are complied with HWE in the control group (*p* > 0.05). The allele frequency of these SNPs had no significant difference between patients and healthy control (rs1879877, *p* = 0.327; rs3181096, *p* = 0.457; rs3181097, *p* = 0.267; and rs3181098, *p* = 0.457).

The complete data of SNP analysis are summarized in Table 4. We found that the polymorphism of rs3181097 was associated with a transfusion reaction (GG vs. AG vs. AA, *p* = 0.003). Additionally, when the rs3181097 with A-allele (AG and AA) had a significantly less chance of transfusion reaction occurrence (OR = 0.287 95% CI = 0.103–0.803, *p* = 0.015). However, there was no significant difference in the genotype frequency of other SNPs (rs1879877, rs3181097, and rs3181098) between the patient group and the healthy control group.

We further analyzed the function of these promoter SNPs with dual-spectral luciferase reporter assay. Firstly, the promoter sequence from −1232 to +200 of the 5’ untranslated region upstream was inserted into a vector expressing luciferase (pnl1.1) by gene cloning. Then, the promoter reporters of rs1879877 G > T, rs3181096 C > T, rs3181097 G > A and rs3181098 G > A were separately constructed by using site-directed mutagenesis. After confirming the sequence of colonization, these vectors were transfected into K562 cells and cultured for 48 h. Subsequently, dual-spectral luciferase reporter assay was used to analyze the impact on the expression of luciferase by different promoter vectors.

The plasmid vector of Firefly was used as an internal control to reduce the deviation of transfection efficiency caused by experimental error. Comparing to the negative control, when the reporter was added, the luminous value increased by more than 10,000-fold. We corrected the relative light unit (RLU) of wild type as the reference, and then compared the data between the wild type and these four promoter reporters (rs1879877 G > T, rs3181096 C > T, rs3181097 G > A and rs3181098 G > A) (Table 5). We found that the RLU of rs3181097 G > A had a significant decrease compared to the wild type (*p* = 0.002), while there was no significant difference in the other three SNPs (rs1879877, rs3181096, and rs3181098) (Table 5).

## 4. Discussion

Although there have been studies focusing on the correlation between SNPs and dis-eases, there are only few studies that further explore the biological function of those SNPs. The present study is the first functional study of SNPs in the CD28 promoter region to explore whether the SNPs located in the promoter region directly affect the level of CD28 expression. Our previous study found that the CD28 genotype was associated with transfusion reactions after a leukoreduced blood product transfusion [6]. We herein continued to expand the number of patients in the case control study, and rs3181097 with A-allele was shown to have a significantly lower risk of transfusion reactions.

Moreover, rs3181097 G > A negatively affected the expression of CD28 in the present luminescence functional study. The principle of the assay was to infer the effect of the promoter gene variation on protein expression according to the intensity of luminescence emitted by the luciferase produced from K562 cells. In these four promoter SNPs of CD28, we found that only rs3181097 affected the intensity of luminescence. It was demonstrated that the expression of CD28 in the minor A-allele of SNP rs3181097 was significantly lower than that in the major G-allele. Combining the results of the case control study and functional analysis, we confirmed that subjects with A-alleles of rs3181097 would have lower risks of transfusion reactions, and the expression level of CD28 was decreased under the effect of A-allele of rs3181097, which would reduce T cell activation and immune response [19].

The transcription factors (TF), Sp1 and c/EBP beta could bind to the DNA sequence with the A-allele of rs3181097 [14], and TFs have been known to could increase or decrease the activity of transcription [20]. Therefore, the decrease in CD28 expression might be caused by the binding of SP1 and/or c/EBP beta to the A-containing sequence of rs3181097. TF is a protein that can bind to a specific DNA sequence. Its function is to initiate and regulate the transcription process of genes, so as to ensure that they are expressed in the correct cells at the appropriate time and in the right amount throughout the entire life of cells and organisms [21]. However, TFs only affect transcription activity (mRNA level), and using mRNA levels together with estimated gene-specific translation rates would overestimate the prediction of protein levels [22]. There are many factors that influence gene expression; therefore, miRNA, alternative splicing, regulation of ribosomes, and eukaryotic initiation factor-2 should also be taken into consideration [23]. Therefore, the finding of rs3181097 G > A negatively affecting the expression of CD28 should be further investigated to confirmed that the promoter SNP is the only factor that affects CD28 expression.

The allele of rs1879877 would determine the TF binding site, where HNF1A could bind to the T-containing sequence of rs1879877 and GATA1 binds to the G-containing sequence [14]. However, our results indicated that it had no significance in RLU between wild type and rs1879877 with T allele. This suggested that rs1879877 might affect the protein expression indirectly. Furthermore, the rs1879877 and rs3181096 had complete linkage disequilibrium (LD) [15], and the location of rs3181097 is very close to these two SNPs. It should be further determined whether there is an LD among these three SNPs. Additionally, the proximal promoter (250 bp) has a greater impact on transcription level than the distal promoter [24]. Therefore, it could explain why rs3181097 had a significant effect on CD28 expression through the proximal promoter [25].

Transfusion reaction is a consequence of immune responses [2], and there are different pathways involving in the immune response, such as the PI3K pathway, grb-2/SOS related p21ras pathway, and ITK/EMT pathway [26,27,28]. In the present study, we only confirmed that CD28 gene polymorphisms are involved in transfusion reactions; other pathways with certain transcription factors or epigenetic modifications should be further explored [29,30].

## 5. Conclusions

In conclusion, we found that the rs3181097 G > A would directly decrease the gene expression level of CD28 and have significantly lower risk in transfusion reactions. This suggests that rs3181097 may play an important role in immune regulation and then lead to the development of transfusion reactions. Furthermore, SNPs, miRNAs, and epigenetic modifications affecting the immune responses should be further investigated in the pathogeneses of diseases.

## Figures and Tables

**Table 1:** The primers for site-directed mutagenesis in CD28 promoter.

**SNP**	**Mutagenesis Primer Sequence**	**SNP** **Position**
rs1879877 (G > T)	F: 5′-TGGAATAACCCTCTCT**T**CAAAGGGCCTGGGA-3′ R: 5′-TCCCAGGCCCTTTG**A**AGAGAGGGTTATTCCA-3′	Promoter -1198
rs3181096 (C > T)	F: 5′-CTCCTTTTGTGCCCTATTA**T**TTAACCTTGAGGG-3′ R: 5′-CCCTCAAGGTTAA**A**TAATAGGGCACAAAAGGAG-3′	Promoter -1106
rs3181097 (G > A)	F: 5′-ACCAAGGGGCTTTTG**A**TTGCCTTACTGTCCCA-3′ R: 5′-TGGGACAGTAAGGCAA**T**CAAAAGCCCCTTGGT-3′	Promoter -1059
rs3181098 (G > A)	F: 5′-GTAACTCCTTTAAAC**A**TTTATGCAGATGTTTCCC-3′ R:5′-GGGAAACATCTGCATAAA**T**GTTTAAAGGAGTTAC-3′	Promoter -820

SNP: single nucleotide polymorphism; SNP position reference PMID: 22977635; F: forward primer; R: reverse primer. The under line of mutagenesis primer sequence was referred to the position of site-directed mutagenesis.

**Table 2:** Characteristics of patients (*n* = 40) with transfusion reaction after transfusing leukoreduced blood products.

	No. (%)
Average age of patients	54.7
Gender (female:male)	30:10
Blood type	
A type	10 (25)
B type	9 (22.5)
AB type	3 (7.5)
O type	18 (45)
Type of blood products	
Leukocyte-poor platelet	19 (47.5)
Leukocyte-poor RBC	21 (52.5)
Type of transfusion reaction	
Febrile non-hemolytic transfusion reaction (FNHTR)	29 (72.5)
Allergic reaction	11 (27.5)

**Table 3:** Allele frequencies in patients and controls and odds ratio for transfusion reaction.

SNP	Allele	Minor Allele Frequency		HWE *p*-Value	Odds Ratio (95% CI.)	*p*-Value ^a^
		Patient	Control			
rs1879877	G/T	0.413	0.338	0.952	1.378 (0.725–2.621)	0.327
rs3181096	C/T	0.263	0.213	0.983	1.319 (0.635–2.741)	0.457
rs3181097	G/A	0.425	0.488	0.085	0.703 (0.377–1.311)	0.267
rs3181098	G/A	0.263	0.213	0.983	1.319 (0.635–2.741)	0.457

HWE: Hardy–Weinberg equilibrium; 95% CI: 95% confidence interval; ^a^ *p*-values were calculated from a chi-squared test for allele frequency. The odds ratio was calculated by taking the minor allele of the patient group as the risk allele.

**Table 4:** Genotypes of CD28 SNPs and their correlations with risk of transfusion reaction.

SNP	Genotype	Patient	Control	Model	Odds Ratio (95% CI)	*p*-Value
		*n* = 40	*n* = 40			
rs1879877	GG	10	5	A		0.328
	GT	13	17	D	1.107 (0.457–2.679)	0.822
	TT	17	18	R	2.333 (0.718–7.587)	0.152
rs3181096	CC	24	25	A		0.479
	CT	11	13	D	1.111 (0.452–2.733)	0.818
	TT	5	2	R	2.714 (0.494–14.901)	0.432
rs3181097	GG	17	6	A		0.003 *
	AG	12	27	D	0.287 (0.103–0.803)	0.015 *
	AA	11	7	R	2.149 (0.707–6.530)	0.172
rs3181098	GG	24	25	A		0.479
	AG	11	13	D	2.715 (0.494–14.901)	0.432
	AA	5	2	R	1.111 (0.452–2.733)	0.818

“*” in *p*-value means that there was a significant difference between patients and healthy controls, *p* < 0.05; 95% CI: 95% confidence interval; A: additive model (AA vs. Aa vs. aa); D: dominant model (AA vs. Aa + aa); R: recessive model (AA + Aa vs. aa).

**Table 5:** The relative light units of the four promoter reporters in each of the 5 tests.

	Test 1	Test 2	Test 3	Test 4	Test 5	Average	*p*-Value
Wild Type	1	1	1	1	1	1	ref
rs1878877G > T	0.85	0.98	0.91	1.1	0.98	0.964	0.438
rs3181096C > T	1.05	1.11	0.86	1.17	0.85	1.008	0.908
rs3181097G > A	0.8	0.86	0.68	0.81	0.74	0.778	0.002 *
rs3181098G > A	0.75	1.18	0.7	1.08	0.75	0.892	0.336

“*” means *p* < 0.05.

## Data Availability

Data available in a publicly accessible repository.

## References

[1] Blood Transfusions for People with Cancer American Cancer Society. https://www.cancer.org/treatment/treatments-and-side-effects/treatment-types/blood-transfusion-and-donation/what-are-transfusions.html..

[2] Dean L. (2005). Blood Groups and Red Cell Antigens [Internet].

[3] Ortolano G.A., Russell R.L., Angelbeck J.A., Schaer J., Wenz B. (2004). Contamination control in nursing with filtration: Part 2: Emerging rationale for bedside (final) filtration of prestorage leukocyte-reduced blood products. J. Infus. Nurs..

[4] Mempel W., Böck M. (1993). Substitution of thrombocyte concentrates in polytransfused patients. Beitr. Infusionsther..

[5] Rajesh K., Harsh S., Amarjit K. (2015). Effects of Prestorage Leukoreduction on the Rate of Febrile Nonhemolytic Transfusion Reactions to Red Blood Cells in a Tertiary Care Hospital. Ann. Med. Health Sci. Res..

[6] Chen D.P., Lin W.T., Wang W.T., Chiueh T.S. (2020). The Influence of CD28 Gene Polymorphism in Transfusion Reaction after Transfusing Leukoreduced Blood Components. J. Clin. Med..

[7] Wen Y.H., Lin W.T., Wang W.T., Chiueh T.S., Chen D.P. (2019). Association of CTLA4 Gene Polymorphism with Transfusion Reaction after Infusion of Leukoreduced Blood Component. J. Clin. Med..

[8] Janeway C.A., Travers P., Walport M., Shlomchik M.J. (2001). Chapter 8: T Cell-Mediated Immunity. Immunobiology: The Immune System in Health and Disease.

[9] Budd R.C., Fortner K.A., Firestein G.S., Budd R.C., Gabriel S.E., McInnes I.B., O’Dell J.R. (2017). T Lymphocytes. Kelley and Firestein’s Textbook of Rheumatology.

[10] Zajac A.J., Blattman J.N., Murali-Krishna K., Sourdive D.J., Suresh M., Altman J.D., Ahmed R. (1998). Viral Immune Evasion Due to Persistence of Activated T Cells without Effector Function. J. Exp. Med..

[11] Sharpe A.H. (2009). Mechanisms of costimulation. Immunol. Rev..

[12] Porciello N., Tuosto L. (2016). CD28 costimulatory signals in T lymphocyte activation: Emerging functions beyond a qualitative and quantitative support to TCR signaling. Cytokine Growth Factor Rev..

[13] Mikhaylichenko O., Bondarenko V., Harnett D., Schor I.E., Males M., Viales R.R., Furlongl E.E.M. (2018). The degree of enhancer or promoter activity is reflected by the levels and directionality of eRNA transcription. Genes Dev..

[14] Kim H.J., Chung J.H., Kang S., Kim S.K., Cho B.S., Kim S.D., Hahn W.H. (2011). Association of CTLA4, CD28 and ICOS gene polymorphisms with clinicopathologic characteristics of childhood IgA nephropathy in Korean population. J. Genet..

[15] Ferjeni Z., Bouzid D., Fourati H., Stayoussef M., Abida O., Kammoun T., Hachicha M., Penha-Gonçalves C., Masmoudi H. (2015). Association of TCR/CD3, PTPN22, CD28 and ZAP70 gene polymorphisms with type 1 diabetes risk in Tunisian population: Family based association study. Immunol. Lett..

[16] Wang G., Sun J. (2017). Interactive Effects of Snps Located Within CD28/B7Pathway and Environment on Susceptibility to Recurrent Spontaneous Abortion. Cell Physiol. Biochem..

[17] Tupikowski K., Partyka A., Kolodziej A., Dembowski J., Debinski P., Halon A., Zdrojowy R., Frydecka I., Karabon L. (2015). CTLA-4 and CD28 genes’ polymorphisms and renal cell carcinoma susceptibility in the Polish population—A prospective study. Tissue Antigens.

[18] Devin M. (2018). Associations between Genetic Variation in the T-Cell Signaling Pathways and CD^4+^ T-Cell Count Recovery after ART Initiation in Southern African Cohort. Master’s Thesis.

[19] Beyersdorf N., Kerkau T., Hünig T. (2015). CD28 co-stimulation in T-cell homeostasis: A recent perspective. Immunotargets Ther..

[20] Adcock I.M., Caramori G., Barnes P., Drazen J., Thomson N. (2009). Chapter 31: Transcription Factors. Asthma and COPD.

[21] Mitsis T., Efthimiadou A., Bacopoulou F., Vlachakis D., Chrousos G.P., Eliopoulos E. (2020). Transcription factors and evolution: An integral part of gene expression (Review). World Acad. Sci. J..

[22] Fortelny N., Overall C.M., Pavlidis P., Freue G.V.C. (2017). Can we predict protein from mRNA levels?. Nature.

[23] Science/Biology Library/Gene Regulation/Gene Regulation in Eukaryotes/Regulation after Transcription. Khan Academy. https://www.khanacademy.org/science/biology/gene-regulation/gene-regulation-in-eukaryotes/a/regulation-after-transcription..

[24] Chen Q.Y., Jackson N. (2004). Human CD1D gene has TATA boxless dual promoters: An SP1-binding element determines the function of the proximal promoter. J. Immunol..

[25] Haberle V., Stark A. (2018). Eukaryotic core promoters and the functional basis of transcription initiation. Nat. Rev. Mol. Cell Biol..

[26] Garcon F., Patton D.T., Emery J.L., Hirsch E., Rottapel R., Sasaki T., Okkenhaug K. (2008). CD28 provides T-cell costimulation and enhances PI3K activity at the immune synapse independently of its capacity to interact with the p85/p110 heterodimer. Blood.

[27] Schneider H., Cai Y.C., Prasad K.V., Shoelson S.E., Rudd C.E. (1995). T cell antigen CD28 binds to the GRB-2/SOS complex, regulators of p21ras. Eur. J. Immunol..

[28] Marengère L.E., Okkenhaug K., Clavreul A., Couez D., Gibson S., Mills G.B., Mak T.W., Rottapel R. (1997). The SH3 domain of Itk/Emt binds to proline-rich sequences in the cytoplasmic domain of the T cell costimulatory receptor CD28. J. Immunol..

[29] Landolin J.M., Johnson D.S., Trinklein N.D., Aldred S.F., Medina C., Shulha H., Weng Z., Myers R.M. (2010). Sequence features that drive human promoter function and tissue specificity. Genome Res..

[30] Carey L.B., van Dijk D., Sloot P.M., Kaandorp J.A., Segal E. (2013). Promoter sequence determines the relationship between expression level and noise. PLoS Biol..

